# Transcriptional regulation of innate lymphoid cells and T cells by aryl hydrocarbon receptor

**DOI:** 10.3389/fimmu.2023.1056267

**Published:** 2023-03-28

**Authors:** Eric Y. Helm, Liang Zhou

**Affiliations:** Department of Infectious Diseases and Immunology, College of Veterinary Medicine, University of Florida, Gainesville, FL, United States

**Keywords:** aryl hydrocarbon receptor, helper T cell, innate lymphoid cell, transcriptional regulation, chromatin, epigenetics

## Abstract

The aryl hydrocarbon receptor (Ahr) is a ligand-dependent transcription factor and facilitates immune cell environmental sensing through its activation by cellular, dietary, and microbial metabolites, as well as environmental toxins. Although expressed in various cell types, Ahr in innate lymphoid cells (ILCs) and their adaptive T cell counterparts regulates essential aspects of their development and function. As opposed to T cells, ILCs exclusively rely on germ-line encoded receptors for activation, but often share expression of core transcription factors and produce shared effector molecules with their T cell counterparts. As such, core modules of transcriptional regulation are both shared and diverge between ILCs and T cells. In this review, we highlight the most recent findings regarding Ahr’s transcriptional regulation of both ILCs and T cells. Furthermore, we focus on insights elucidating the shared and distinct mechanisms by which Ahr regulates both innate and adaptive lymphocytes.

## Introduction

Immune cells are constantly sensing and reacting to molecular signals provided by the environment, commensal microflora, diet, and host metabolism. Multiple sensors exist to detect and integrate these signals into cellular responses. The aryl hydrocarbon receptor (Ahr) is one such ligand-dependent sensor that is activated in response to chemicals derived from host metabolism, diet, commensal flora and the environment [reviewed in (1)]. Xenobiotic and non-xenobiotic Ahr ligands have been extensively described elsewhere [reviewed in (2–4)]. In its inactive form, Ahr remains in the cytosol in complex with chaperon proteins including aryl hydrocarbon interacting protein (AIP) (5, 6), prostaglandin E synthase 3 (PTGES3) and heat-shock protein 90 kDa (HSP90) (7, 8). Upon ligand binding, Ahr translocates to the nucleus and dimerizes with its binding partner aryl hydrocarbon receptor nuclear translocator (Arnt) and can directly bind to DNA to regulate target gene expression [reviewed in (9)]. Aspects of Ahr biology including its structure, the metabolic pathways and generation of ligands that activate Ahr have been reviewed elsewhere [reviewed in (2, 4, 10, 11)]. In brief, Ahr was initially identified as a sensor of the xenobiotic chemical 2,3,7,8-tetrachlorodobidenzo-p-dioxin (TCDD) (12). Since then, multiple Ahr ligands, including those derived from tryptophan (Trp) metabolism and the microbiota have been identified. Kynurenine (Kyn) is an Ahr ligand resulting from Trp metabolism by indoleamine-2,3-dioxygenase (IDO) and tryptophan-2,3-dioxygenase (TDO) (13, 14). IDO/TDO independent metabolism of Trp can also generate Ahr ligands (15). In addition, photo-oxidation as well as metabolic pathways can convert Trp into 6-formylindolo[3,2-b]carbazole (FICZ), another Ahr agonist (16, 17). Microbiota-derived Ahr ligands have also been described, including the production of indole-3-aldehyde (I3A) by *Lactobacilli *via Trp catabolism (18). In addition, Ahr agonist precursors are found in high abundance from the diet, including from the *Brassica* genus, in which the precursors are metabolized into indole-3-carbinol (I3C) and indole-3-acetonitrile (I3ACN). I3C and I3CAN are subsequently converted into Ahr ligands including 3,3’ diindolylmethane (DIM) [reviewed in (4)].

Ahr is an essential component in facilitating the integration of environmental signals in the host immune system response including in T cells and innate lymphoid cells (ILCs) [reviewed in (4, 9)]. ILCs, which rely on germ-line encoded receptors for activation [reviewed in (19)], resemble CD4^+^ and CD8^+^ T cell subsets in their functional and developmental programs [reviewed in (20)]. Thus, while clear differences exist between ILCs and their T cell counterparts, aspects of their core transcriptional programming are shared. One such shared aspect between ILCs and certain T cell subsets is the expression of Ahr [reviewed in (4, 9)]. However, Ahr acts in a complex network of transcription factors. This network changes in a cell-type and organ specific manner and ultimately guides cellular outcomes in response to different stimuli. Understanding how Ahr integrates into these networks is critical for elucidating the underlying transcriptional mechanisms by which immune cells are regulated. In this review, we discuss the current knowledge regarding Ahr’s transcriptional regulation of T cells and ILCs and the shared and unique mechanisms by which Ahr regulates those cells.

## Considerations of cell intrinsic and extrinsic effects

The broad expression pattern of Ahr, including in both immune and non-immune cells, makes consideration of possible cell-intrinsic and extrinsic effects of Ahr essential in experimental design. Systemic Ahr ligand administration, in which Ahr ligands are administered *via* oral or intraperitoneal routes to mice, can non-specifically activate Ahr in multiple cell types. In these experiments, solubilized Ahr ligands are administered in single or multiple doses, and subsequent cellular changes are assessed days to weeks later and can lead to cell-extrinsic impacts on immune cells. For instance, systemic administration of the Ahr ligand TCDD impaired CD8^+^ T cell primary and secondary responses to influenza (21). However, this defect was ultimately attributed to the impact of TCDD on other immune cells affecting CD8^+^ T cell priming. Similarly, germ-line deletion of Ahr in mice makes interpretation of cell-intrinsic effects of Ahr deletion murky. Conflicting studies on the role of Ahr in regulatory T cells (Treg) utilized complete Ahr-null mice or systemic TCDD administration, highlighting the complexity of interpreting experimental systems that non-specifically modulate Ahr (22–26) (**Table 1**). Further adding to the complexity of systemic ligand administration, different Ahr ligands have been reported to have divergent effects on immune cells. For example, FICZ promotes Th17 cell differentiation *in vitro*, while kynurenine does not (26, 32, 59). Further, FICZ appears to inhibit TGFβ-induced Treg cells *in vitro*, while kynurenine supports their differentiation (13). In addition, the concentration of exogenously administered Ahr ligands leads to differential effects on murine CD4^+^ T cell differentiation (60). To this end, utilizing genetic models, cell-type specific rescuing experiments and bone marrow chimeras to definitively tease apart cell-intrinsic and -extrinsic effects of Ahr are essential. As such, this review will focus on cell-intrinsic effects of Ahr when possible.

**Table 1:** Cell type specific, cell intrinsic effects of Ahr in ILCs and T cells.

Cell type	Ahr effect	Cell-intrinsic regulation	Reference
**Treg**	Promotes intestinal homing. Suppresses inflammatory phenotype	Yes	(27–29)
	Promotes differentiation	Not determined	(22, 23, 25, 26)
	Suppresses differentiation	Not determined	(24, 26)
**Tr1**	Promotes differentiation, intestinal homing	Not determined	(22, 30, 31)
**Th17/Th22**	Promotes Th17 or Th22 differentiation	Not determined	(26, 32–34)
**CD8^+^**	Promotes intestine T_RM_ differentiation, anti-tumor response	Yes	(35)
	Promotes T_RM_ differentiation	Not determined	(36)
	Promotes anti-tumor response	Not determined	(37, 38)
	Inhibits anti-tumor response	Not determined	(21, 39, 40)
**γδ T cell**	Promotes differentiation in skin, intestine	Yes	(41)
	Promotes differentiation, survival, and effector function	Not determined	(42–46)
**ILC1**	No effect on differentiation. Promotes NK effector function and anti-tumor response	Yes	(47, 48)
	Promotes IL-10 and IFNγ production in NK cells	Not determined	(49)
	Promotes ILC1 maintenance, differentiation	Not determined	(50, 51)
**ILC2**	Restricts effector function. Dispensable for differentiation	Yes	(52)
	Promotes effector function	Not determined	(53, 54)
**ILC3**	Promotes maintenance and effector function	Yes	(55–58)

## Ahr in CD4^+^ T cells

In murine T helper (Th) cells, Ahr is highly expressed in Treg, Th17 and Th22 cells, and is only minimally expressed in Th1, Th2 and naïve T cells (30, 32). However, it is worthy of note that these expression patterns of Ahr are derived from the data using *in vitro* differentiation assays and Ahr expression *in vivo* may differ (32). Thus, more careful examination of Ahr’s expression and potential function in Th1 and Th2 cells is warranted. As such, this review will focus on the role of Ahr in regulating Treg, Th17 and Th22 cell identity and function.

### Ahr in Treg cells

Foxp3^+^ Treg and Foxp3^–^ IL-10 producing Tr1 cells are essential for maintaining immune homeostasis. While Foxp3 is essential for maintaining Foxp3^+^ Treg cell identity, other transcription factors are also expressed and contribute to Treg cell differentiation and function [reviewed in (61)]. Recent studies using genetic or experimental approaches have elucidated both the expression and cell intrinsic function of Ahr in murine Treg cells (27–29). Utilizing a Foxp3^+^ Treg-cell specific Ahr reporter mouse, Ahr expression was found to be highest in Treg cells in the small and large intestines but at lower levels in other lymphoid and non-lymphoid tissues (27). A Treg cell-specific deletion of *Ahr* revealed multiple roles for Ahr in Treg cell function. Ahr was found to be dispensable for Foxp3 expression, which is further corroborated by the lack of alterations in CpG methylation or chromatin accessibility at the *Foxp3* locus. Furthermore, Foxp3-cre fate mapping studies revealed that Ahr-deficient Treg cells maintained Foxp3 expression to equal extents as control Treg cells. In addition, no global alterations in transcriptional programs were observed in Ahr-deficient Treg cells. Rather, genetic ablation of *Ahr* solely in Treg cells revealed that only a small set of genes were dependent on Ahr in Treg cells at least under steady state. Ahr was essential for accumulation of gut-Treg cells at steady state by promoting expression of gut-homing receptors including *Gpr15* in concert with Foxp3 (28, 29). Furthermore, while Treg effector genes such as *Ctla4, Entpd1*, and *Nt5e* were unaltered, Ahr-deficient Treg cells aberrantly expressed pro-inflammatory cytokines including IFNγ and IL-17A and failed to suppress T-cell mediated colitis (27). Collectively these studies demonstrate a cell-intrinsic role for Ahr in promoting Treg cell intestinal homing and suppressive function.

### Ahr in Tr1 cells

In contrast to Foxp3^+^ Treg cells, Tr1 cells lack Foxp3 expression but still produce high amounts of the suppressive cytokine IL-10 (62). Adoptive transfer of CD4^+^ T cells isolated from mice with reduced Ahr ligand binding affinity (Ahr^d^ mice) that were polarized under Tr1 conditions *in vitro* led to significantly enhanced experimental autoimmune encephalomyelitis (EAE) as compared to mice receiving wild type CD4^+^ T cells polarized in Tr1 conditions. Furthermore, Ahr^d^ mice exhibited reduced Tr1 cell differentiation at steady state (30). While these results suggest a cell intrinsic role for Ahr in promoting Tr1 cell differentiation, it remains a possibility that cell extrinsic factors may predispose CD4^+^ T cells in Ahr^d^ mice towards a more inflammatory state, and that *in vitro* polarized Tr1 cells may not reflect bona fide Tr1 cells generated *in vivo. In vitro* evidence also supports a role for Ahr in driving Tr1 cell effector function in both mice and humans (22, 30, 63). Electrophoretic mobility shift assays in both mouse and human Tr1 cells revealed Ahr binding to the *Il10* promoter, suggesting direct regulation of *Il10* by Ahr (22, 30). Like its role in Foxp3^+^ Treg cells, Ahr has been implicated in promoting Tr1 cell intestinal homing (31). In an alloreactive adoptive transfer system, systemic administration of recipient mice with TCDD significantly increased expression of gut-homing receptor Ccr9 and enhanced Tr1 cell migration to both the small and large intestine (31).

### Ahr in Th17/22 cells

Th17 cells require expression of the transcription factor RORγt (64) and produce effector molecules including IL-17A, IL-17F, IL-22, and GM-CSF [reviewed in (65)]. Expression of Ahr in Th17 cells is driven by external stimuli, including cytokines such as IL-6, IL-21 and TGFβ (9, 32, 59), and activation of Ahr *in vitro* by ligands enhances Th17 cell differentiation in both mice and humans (26, 32, 59, 66) as well as production of IL-17A and IL-22 (33, 59). IL-2 inhibits Th17 cell differentiation (67, 68) and Ahr has been shown to limit IL-2 production in murine Th17 cells (34). Genetic ablation of Ahr by germline deletion is associated with reduced pathogenicity of Th17 cell mediated diseases, including EAE in mice (32), and other studies have demonstrated that Ahr activation by systemic TCDD administration can exacerbate EAE as well (26).

While IL-22 can be produced by Th17 cells, some CD4^+^ T cells only express IL-22 and not IL-17A and provide protection against enteric pathogens including *Citrobacter rodentium* (9, 33, 69, 70). These Th22 cells can be induced solely by IL-6 or IL-21 in combination with TCR stimulation (33, 71, 72). Conversely, TGFβ, which enhances IL-17A production by CD4^+^ T cells (73), appears to inhibit IL-22 production in Th22 cells (71). Expression of Ahr is lower in murine Th22 cells as compared to Th17 cells, consistent with the observation that TGFβ and IL-6 synergistically induce its expression. However, Ahr is essential for IL-22 production in both populations (26, 33). As such, Ahr-deficient Th22 cells failed to efficiently protect against *Citrobacter rodentium* infection and to limit T cell-mediated colitis in mice (33, 72).

## Ahr in CD8^+^ T cells

Given the higher expression of Ahr in CD8^+^ T cells in non-lymphoid tissues including the skin and lung (36, 37), a recent study utilizing an oral *Listeria monocytogenes* infection model investigated the cell intrinsic role for Ahr in resident-memory (T_RM_) cells (35). In an adoptive co-transfer system with antigen specific CD8^+^ T cells, Ahr was found to be essential for intestinal T_RM_ cell differentiation. The defect in T_RM_ cell differentiation could be attributed to impaired accumulation of T_RM_ cell precursors early during the differentiation process, suggesting that Ahr regulates early lineage decision processes to promote T_RM_ cell differentiation. Furthermore, Ahr*-*deficient T_RM_ cells produced less of the effector molecule Granzyme B (35). Of note, using a *Cd8*-driven Cre recombinase-mediated *in vivo* deletion approach in mice, Ahr was also shown to regulate gut CD8^+^ T_RM_-like cells by promoting a T_RM_-like transcriptional program under the physiological conditions (35). These data are consistent with the observation that the gut milieu represents a unique “inflammation state” that is continuously exposed to various commensals and dietary stimuli to provide Ahr ligands, and the highest expression of Ahr in gut T_RM_-like cells among CD8^+^ T cell populations in mice (35). A separate study found that Ahr was highly expressed in murine skin T_RM_ cells following HSV infection, and Ahr-deficient CD8^+^ T cells failed to persist in the skin in response to 1-Fluoro-2,4-dinitrobenzene (DNFB) mediated skin-inflammation (36). Collectively, these studies demonstrate a cell-intrinsic role for Ahr in promoting T_RM_ cell differentiation.

The cell intrinsic role of Ahr in anti-tumor responses by CD8^+^ T cells has recently been investigated (35). Using both B16F10 melanoma and MC38 colon carcinoma models, Ahr was found to promote CD8^+^ tumor infiltrating lymphocyte (TIL) effector function. Further, mice in which Ahr was ablated solely in CD8^+^ T cells had substantially higher tumor burden as compared to control mice. Thus, this study supports Ahr promoting CD8^+^ T cell anti-tumor responses in a cell-autonomous manner (35). Other studies have also suggested that Ahr may promote anti-tumor responses by CD8^+^ T cells. Ahr activation through systemic administration of FICZ activated IL-22 producing CD8^+^ T cells (Tc22 cells), which have enhanced anti-tumor effects, though the cell extrinsic effect of FICZ on other cell populations remains possible (38). The precise mechanisms by which Ahr regulates CD8^+^ TILs are elusive, and whether Ahr supports CD8^+^ TILs in response to other cancers is an open question. Consideration for the availability of Ahr ligands in different tumor environments is essential. For example, expression of IDO, which converts tryptophan into the Ahr ligand kynurenine, varies among different human and mouse cancers (74), suggesting that differing concentrations of Ahr ligands may exist among various cancer types. Further, as Ahr expression varies in tissue CD8^+^ T cells (35), Ahr may have differential effects on CD8^+^ TIL effector function in differing tumor-bearing tissues as well. Finally, certain studies relying on systemic Ahr ligand administration to interrogate anti-tumor CD8^+^ T cell responses implicate Ahr in promoting CD8^+^ T cell dysfunction (39, 40). However, these seemingly discrepant conclusions (**Table 1**) may reflect cell-extrinsic effects of Ahr activation on CD8^+^ T cell anti-tumor responses.

## Ahr in γδ T cells

γδ T cells are found in high abundance particularly in non-lymphoid tissues including the skin, liver, lungs and intestines [reviewed in (75)]. Compared with conventional αβ T cells, γδ T cells exhibit less TCR diversity, but do not require activation to gain effector function and instead, can constitutively express effector molecules (76). γδ T cells have been demonstrated to be essential for both regulation of tissue homeostasis and immune responses (41, 42, 77, 78). While Ahr is expressed in murine γδ T cells found both in circulation and in non-lymphoid tissues, its expression is higher in skin and intestine-resident γδ T cells as compared to γδ T cells found in the lymphoid tissues (41). This tissue specificity is consistent with the abundance of Ahr ligands in these tissue milieus. Ahr was particularly important for maintenance of murine γδ T cells in both the skin and intestinal epithelium, in part by sustaining expression of c-Kit, a molecule important for cell proliferation (79), and by promoting γδ T cell survival (41, 43). Notably, in the intestinal epithelium, Ahr cell-intrinsically promoted γδ T cell maintenance, as demonstrated through bone marrow chimera experiments, but was found to be dispensable in secondary lymphoid organs, liver and intestinal lamina propria (41). These distinct requirements for Ahr in the same cell type but different tissues was also observed in Tregs (27). The underlying mechanisms by which Ahr differentially regulates the maintenance of γδ T cells in different organs remain unclear, and are unlikely to be simply explained by lack of Ahr expression in those tissues, as Ahr is still expressed in systemic γδ T cells as well (41). As noted earlier, different Ahr ligands having divergent effects on Th17 and Treg cells (13, 26, 32, 59) and the concentration of exogenously administered Ahr ligands leads to differential effects on murine CD4^+^ T cell differentiation. This raises the possibility that differing Ahr ligand abundance in tissues may contribute to organ-specific regulation of γδ T cells by Ahr (60). However, the specific quantification and functional effect of different Ahr ligands on γδ T cells *in vivo* may not be feasible with current tools available. In addition, the nature of Ahr interacting partners may differ between γδ T cells in different organs. Leveraging new advances in low cell input mass spectrometry and other single cell genetic approaches may help elucidate the nature of Ahr interacting partners in an organ-specific manner (80, 81).

Like its role in other IL-22 producing immune cells, Ahr is essential for IL-22 production in both murine and human γδ T cells as well (42, 44, 82). In the skin, Ahr-mediated regulation of IL-22 production in human and mouse γδ T cells is dependent on CD69 expression, which promotes the uptake of the Ahr ligand FICZ through stabilization of the amino acid transporter complex LAT1-CD97 (44). Germline Ahr-deficient mice have enhanced susceptibility to dextran sodium sulfate (DSS)-mediated colitis and impaired intestinal epithelial repair. This enhanced susceptibility to DSS-colitis could be rescued by the adoptive transfer of wild type intraepithelial lymphocytes (IEL), which contain γδ T cells (41). These results suggest that Ahr plays an essential protective role in IEL response to DSS-mediated colitis. However, the rescuing effect of wild type IEL reconstitution to germline Ahr-deficient mice is not necessarily due to restoration of γδ T cells, as other immune cells present in the IEL compartment, such as TCRαβ^+^ CD8αα^+^ cells, can prevent CD4^+^ T cell mediated colitis (83). Other studies additionally implicate Ahr in driving IL-22 production in γδ T cells, including during lung fibrosis, intestinal injury, and psoriasis (42, 45, 46) (**Table 1**). Taken together, these studies implicate Ahr in sustaining IL-22 in γδ T cells in a variety of tissues. However, these studies investigating the role of Ahr in γδ T cell function, and the physiological consequences of Ahr-deficiency in γδ T cells largely relied on genetic systemics in which Ahr is perturbed at the germline level, or utilized systemic administration of Ahr ligands. As such, careful studies utilizing γδ T cell specific deletion of Ahr, such as *Tcrd^CreER^* mediated approaches, are essential to elucidate the role of Ahr in γδ T cell effector function in the context of disease.

## Ahr in ILC1s

Our understanding of the role of Ahr in group 1 ILCs, consisting of NK and ILC1, is limited as compared to our knowledge in other ILC and T cell populations. In human NK cells, differing reports of Ahr expression in immature versus mature NK cells exist (84–86). This discrepancy in Ahr expression may in part be attributed to organ specific differences in Ahr expression, similar to observations in murine Treg cells (27), or due to different assays to determine Ahr expression (qPCR versus protein staining), or variation of human samples. In mice, Ahr expression has been reported to be highest in immature versus mature splenic NK cells (47). Furthermore, murine CD49a^+^ liver NK cells, which may be ILC1 (87), exhibit higher expression of Ahr as compared to the conventional CD49b^+^ NK cells (50) (**Table 1**). As such, further investigation of Ahr expression in both NK and ILC1 cells through both mRNA, protein and reporter assays is essential, particularly in intestinal tissues where Ahr activity tends to be highest (88).

Germline deletion of Ahr had no effect on NK cell numbers and maturation in the spleen and bone marrow, suggesting that Ahr may be dispensable for NK cell ontogeny in mice (47, 50). Nonetheless, Ahr has also been implicated in preferentially supporting ILC1 over NK cell differentiation in both mice and humans (50, 51). Reconstitution assays support a cell intrinsic role for Ahr in promoting a specific subset of TRAIL^+^ liver ILCs, which may encompass both NK and ILC1 (89), while Ahr was dispensable for conventional CD49b^+^ NK cell development (50). In addition, Ahr has also been implicated in preventing differentiation of ILC3 to NK cells in both mice and humans (85, 90, 91). These data support the hypothesis that Ahr may preferentially support differentiation of ILC1 and ILC3 over NK cells. However, the cell intrinsic role for Ahr in regulating NK or ILC1 cell development has yet to be fully elucidated and further clarification using genetic tools to interrogate the specific regulation of NK and ILC1 development and maintenance in different tissues by Ahr will be essential.

While the role of Ahr in NK and ILC1 ontogeny remain opaque, there is evidence that Ahr regulates their function, particularly in NK cells. FICZ treatment of human and murine NK cells enhanced production of IFNγ (47, 84) and murine Ahr-deficient NK cells produced less IFNγ (49). In this line, Ahr ChIP in human NK cells revealed Ahr binding at the :*Ifng* locus, suggesting direct regulation by Ahr (51). Furthermore, murine Ahr-deficient NK cells exhibited reduced killing capacity *in vitro* (47), though human NK cells treated with the Kyn exhibited reduced NK cell cytotoxicity (92) but may reflect species-specific differences in Ahr function. Adoptive transfer experiments in mice support a cell intrinsic role for Ahr in supporting anti-tumor responses of NK cells (47, 48). Whether Ahr similarly regulates ILC1 effector function remains to be determined. Furthermore, given the current evidence suggesting that Ahr may restrict NK cell differentiation while simultaneously promoting their effector function, subsequent mechanistic studies will be essential to clarify this dichotomous phenomenon. Specifically, the epigenetic status of NK cell progenitors versus mature NK cells should be established to determine whether changes in histone modifications and/or chromatin accessibility drive differences in gene regulation by Ahr. Further, biochemical analyses to elucidate different transcription factors that Ahr is in complex with at different stages may further shine light on the potentially differential regulation of NK cell development and function by Ahr.

## Ahr in ILC2s

Group 2 ILCs are found in abundance both in lymphoid and non-lymphoid tissues including the gut, skin, lung, spleen and adipose tissue (93). At steady state, Ahr is expressed at high levels in murine gut-resident ILC2 and is minimally or not expressed in the lung, skin, fat or circulating ILC2 (52). While Ahr-deficient mice had increased numbers of ILC2s in the gut, this was consistent with the corresponding loss of ILC3s in those mice and demonstrates a role for Ahr in balancing the accumulation of ILC2 versus ILC3 in the intestines. However, using mixed bone marrow chimeras, Ahr was found to cell-intrinsically restrict gut ILC2 effector function. Suppression of ILC2 effector function by Ahr was in part due to negative regulation of IL-33 signaling by restricting *Il1rl1* (gene encoding the IL-33 specific receptor subunit ST2) chromatin accessibility and expression. Adoptive transfers of Ahr-deficient ILC2s exhibited enhanced ability to clear helminth infection as compared to WT ILC2s (52) (**Table 1**).

Despite low expression of Ahr at steady state in the lung (52), Ahr was found to have a role in lung ILC2 effector function under certain conditions. Specifically, Ahr regulates the expression of IL-17A in lung ILC2 in both mice and humans (53, 54, 94). In germline Ahr-deficient mice, treatment with IL-33 significantly reduced expression of IL-17A in lung ILC2. Further, *in vitro* culture of sort-purified Ahr-deficient ILC2 with IL-33 reduced IL-17A production, suggesting that Ahr promotes IL-17 expression in lung ILC2 in response to IL-33 (54). Given the ability for Ahr to sustain its own expression in gut-ILC2 (52), it remains a possibility that IL-33 treatment may induce Ahr expression in the lung, which was not observed at steady state. In contrast to its role in restricting IL-33 signaling and type 2 cytokine production in gut ILC2 (52), Ahr deficient in lung ILC2 exhibited no changes in ST2 or IL-5 expression (54), suggesting organ-specific regulation of ILC2 effector function by Ahr. The divergent regulation of ILC2s in different organs likely reflect variable expression of transcriptional regulators in various organs that modulate Ahr activity and its interaction with other binding partners that regulate Ahr function.

## Ahr in ILC3s

Essential functions for Ahr in ILC3s have been previously described (55–58) (**Table 1**). While at the fetal liver stage and in neonatal mice, Ahr is dispensable for lymphoid tissue inducer (LTi) cell development, Ahr is required for ILC3 maintenance and IL-22 production (55, 56). Ahr in part sustains ILC3 maintenance by promoting expression of both anti-apoptotic genes such as *Bcl2*, as well as IL-7 receptor, which is essential for IL-7 signaling to maintain ILC3s. In addition, Ahr drives expression of *Kit* by direct binding to the *Kit* promoter to promote ILC3 proliferation (55). Ahr also promotes postnatal ILC3 differentiation by inducing Notch1 and Notch2, thereby sustaining Notch signaling in ILC3s (57). As a result of its critical role in ILC3 maintenance and function, Ahr is essential for clearance of *Citrobacter rodentium* (55, 56). Using an *Ahr* knock-in genetic approach with a floxed STOP cassette incorporated at the endogenous *Ahr* locus-*Ahr^CAIR^* mice (27), Ahr expression in RORγt^+^ lymphocytes has also been shown to be sufficient for ILC3 maintenance and function during *Citrobacter rodentium* infection (52). These data highlight both the necessary and sufficient role for Ahr in murine ILC3s. Furthermore, Ahr expression in ILC3s restricts Th17 cell differentiation by sustaining IL-22 expression and suppression of Segmented filamentous bacteria (SFB) outgrowth (58). Given the previous observation that antigen-presenting ILC3s restrict Th17 cell differentiation through antigen presentation (95), Ahr expression in ILC3s may suppress Th17 cell differentiation through a similar manner as well.

## Transcriptional control of T cells and ILCs by Ahr

### Ahr and Stat3

**Figure 1:**
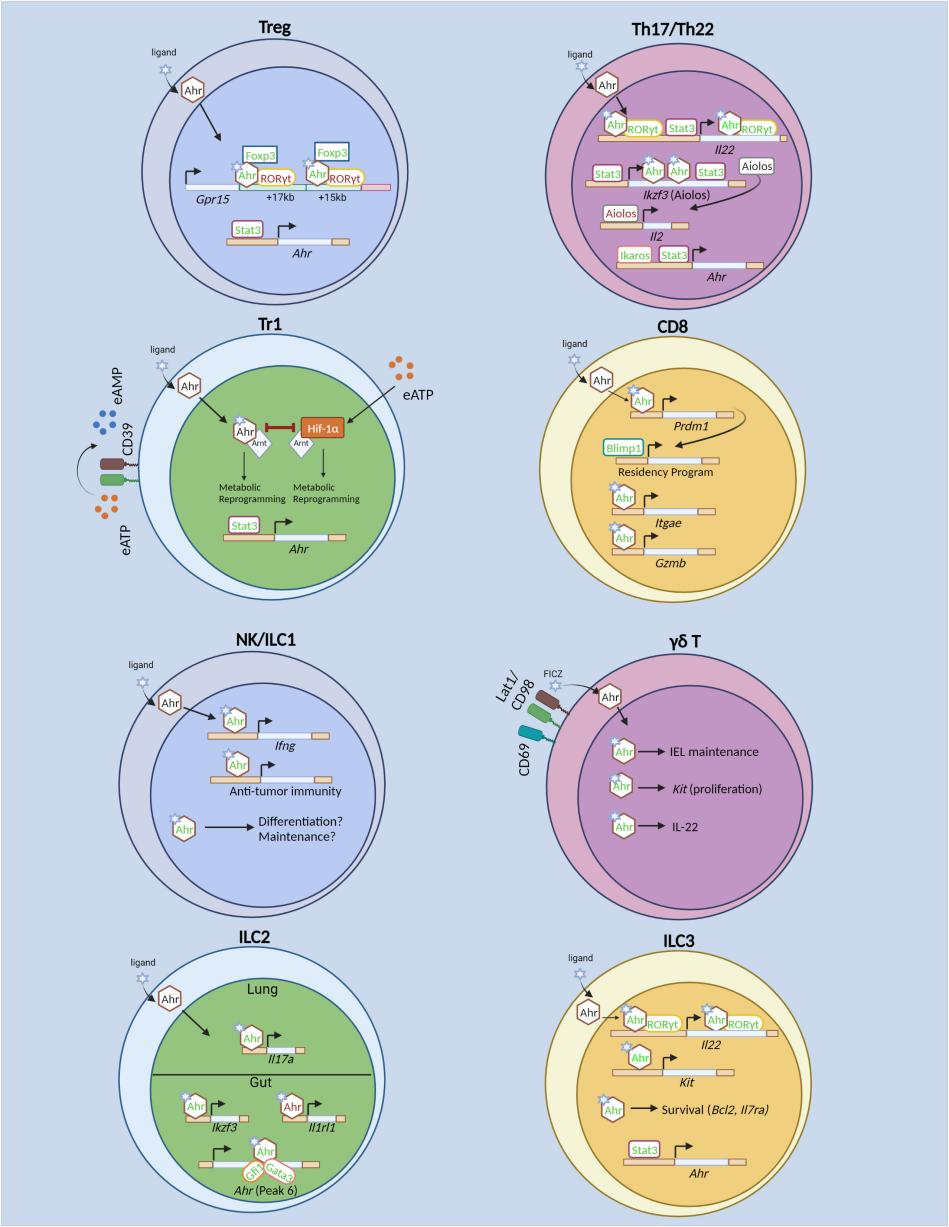
Transcriptional regulation of T cells and innate lymphoid cells by the Aryl hydrocarbon receptor (Ahr). In regulatory T (Treg) cells, Stat3 drives Ahr expression. Ahr directly promotes transcription of gut-homing receptor *Gpr15.* Ahr acts cooperatively with GATA3 and Foxp3 to drive *Gpr15* expression. Specifically, Ahr physically interacts with Foxp3 to promote *Gpr15* expression. Ahr also interacts with RORγt, the latter of which acts in opposition to Ahr to restrict *Gpr15* expression. In Tr1 cells, Ahr and Hif-1α promote glycolysis at different timepoints during differentiation. However, Hif-1α and Ahr antagonize each other as well. Hif-1α is induced by extracellular ATP (eATP). Stat3 promotes Ahr both by direct binding to the *Ahr* locus, as well as driving CD39 expression which converts eATP to AMP, thus limiting Hif-1α-mediated inhibition of Ahr. In Th17 and Th22 cells, Ikaros and Stat3 promote Ahr expression. Ahr cooperates with RORγt and Stat3 to drive *Il22* expression. Ahr and Stat3 promote Th17 cell differentiation by driving expression of *Ikzf3* (Aiolos), which subsequently limits IL-2 production and prevents autocrine IL-2 signaling. In CD8^+^ T cells, Ahr is known to promote T_RM_ differentiation *in vitro* and *in vivo*. Also, it regulates T_RM_ function by promoting *Gzmb* (Granzyme B) expression and enhances polyfunctional CD8^+^ T cells in tumor models. Ahr expression is induced *in vitro* by cytokine stimulation in NK cells and promotes NK cell maintenance. The role for Ahr in NK and ILC1 effector function and differentiation are unknown. Ahr promotes γδ T cell maintenance within the IEL compartment in part by sustaining *Kit* expression and promotes IL-22 expression. However, it is unclear whether similar mechanisms of action of Ahr to promote *Kit* and *Il2*2 transcription as identified in ILC3s remain intact in γδ T cells. In ILC2s, Ahr promotes its own expression in conjunction with Gata3 and Gfi1. In gut-ILC2s, Ahr restricts ILC2 effector function by inhibition of *Il1rl1* (encoding ST2). In lung ILC2s, Ahr sustains *Il17a* (IL-17) production. In ILC3s, Ahr is required for ILC3 proliferation through direct regulation of *Kit* and promotes ILC3 cell proliferation and survival. In addition, Ahr cooperates with RORγt to drive *Il22* expression. In contrast to Th17 cells, Stat3 does not promote Ahr expression in ILC3s, but may cooperate with Ahr to promote *Il22*. Green text indicates transcriptional promotion, while red text indicates transcriptional repression. Created with BioRender.com.

Stat3 regulates the Ahr pathway at multiple levels in T cells. In response to IL-6, Stat3 is activated and can directly bind to the *Ahr* promoter to drive *Ahr* expression during murine Th17 and Tr1 cell differentiation *in vitro* (**Figure 1**) (96, 97). Furthermore, Stat3 has been shown to cooperate with Ahr to modulate target gene expression. The Ikaros family zinc-finger transcription factor *Ikzf3* (encoding Aiolos) is expressed in multiple Th cell subsets and supports both Th17 and Treg cell differentiation (22, 34, 98). During Th17 cell differentiation, Aiolos is essential to limit IL-2 production, thereby preventing autocrine IL-2 signaling and promoting Th17 cell differentiation (34). Both Ahr and Stat3 bind the *Ikzf3* locus and synergistically drive *Ikzf3* expression in luciferase assay (34). Cooperation between Stat3 and Ahr is also essential for IL-22 production in murine Th22 cells (72). *Stat3*-deficient CD4^+^ T cells fail to produce IL-22 in response to IL-21 stimulation and have reduced Ahr expression, suggesting Stat3 may promote Ahr expression in Th22 cells as well. Like their co-regulation of *Ikzf3*, Ahr and Stat3 both bind to conserved and separate regions of the *Il22* locus in Th22 cells. Co-transduction of *Ahr* and *Stat3* was sufficient to drive *Il22* expression in luciferase assay, suggesting coordination between both transcription factors promotes *Il22* transcription. Epigenetically, loss of Stat3 during Th22 cell differentiation led to impaired deposition of active histone modifications at the *Il22* locus and reduced recruitment of Ahr. However, alterations in histone modifications was not observed in *Stat3*-deficient naïve CD4^+^ T, suggesting that Stat3 is involved in epigenetic remodeling of the *Il22* locus during Th22 cell differentiation and facilitates Ahr recruitment to the locus (72). Of note, in murine CD4^+^ T cells where the transcription factor Ikaros’ DNA binding is abrogated, IL-22 is aberrantly produced in an Ahr dependent manner (99). Furthermore, this ablation of Ikaros DNA binding in conjunction with Stat3-deficiency completely abrogated IL-22 production in CD4^+^ T cells and FICZ treatment had no effect on IL-22 (99). As such, we speculate that Stat3 may be a pioneering transcription factor that acts early during Th22 cell differentiation to remodel the *Il22* locus, thereby facilitating IL-22 production. Indeed, Stat3 is able to directly interact with the histone acetyl transferase p300 to promote chromatin remodeling and transcriptional activation in mouse embryonic fibroblasts (100). In addition, Stat3 expression is transcriptionally induced and activated by phosphorylation upon IL-6 and IL-23 stimulation much more rapidly as compared to other transcription factors essential for IL-22 production, including RORγt and Ahr (34), consistent with the notion that it acts as an initiating factor for Th22 differentiation. Kinetic analysis of the epigenetic status of the *Il22* locus through ATAC-seq and histone modification ChIP-seq during Th22 cell differentiation in the presence and absence of Stat3 and Ahr may help elucidate the specific mechanism by which Stat3 and Ahr regulate the *Il22* locus. Of note, Stat3 facilitates chromatin remodeling of the *Il22* locus at regions bound by Ahr, suggesting that Stat3 binding is required for chromatin remodeling of Ahr-bound regions (72). In addition, RORγt is required for Ahr binding to those specific regions (72). As such, Stat3 may interact with RORγt, as is observed in transfected HeLa cells (101), which may subsequently facilitate the chromatin remodeling and recruitment of Ahr to the *Il22* locus.

The regulation of ILC3s by Stat3 and Ahr may differ as compared that of Th17 and Th22 cells. When Stat3 is ablated in RORγt^+^ lymphocytes, mice rapidly succumbed to *Citrobacter rodentium* infection in an IL-22-dependent manner (102). Furthermore, both murine T cells and ILCs exhibit significantly reduced IL-17A and IL-22 production in the absence of Stat3 *in vivo*. However, only the differentiation of RORγt^+^ T cells was reduced in the absence of Stat3, while ILC3 differentiation was unaltered, demonstrating that Stat3 is dispensable for ILC3, but not Th17 cell differentiation (**Figure 1**) (102). Furthermore, while Stat3 promoted *Ahr* expression in murine Th17 and Th22 cells (72, 96), Ahr expression was unaltered in Stat3-deficient ILC3s (102). As such, Stat3 may not drive *Ahr* expression in ILC3s, though the specific mechanisms by which Stat3 regulates Ahr expression in ILC3s versus Th17/Th22 cells are unclear. The differences may simply be attributed to the fact that the Th17 and Th22 cell data were derived from *in vitro* differentiated cells, while ILC3s were sort-purified *ex vivo.* Similar to its role in Th17 and Th22 cells (33, 96), Stat3 was essential for IL-22 production in murine ILC3s and bound to the *Il22* locus in primary ILC3s (102). Whether Ahr and Stat3 also cooperate to drive *Il22* expression in ILC3s remains an open question. Furthermore, whether the cooperative relationship of Ahr and Stat3 observed in Th17 and Th22 cells extend to other genomic loci remains to be determined. As such, there are both conserved and non-conserved aspects of Ahr and Stat3 regulation of innate versus adaptive immune cells.

### Ahr and RORγt

Ahr and RORγt act cooperatively to drive IL-22 expression in both ILC3s and T cells (**Figure 1**). When co-expressed in EL4 cells, Ahr and RORγt synergistically drive IL-22 expression (56). This cooperation was observed in primary murine T cells as well, as RORγt deficiency led to reduced Ahr recruitment to the *Il22* locus upon IL-21 stimulation (72). Ahr and RORγt were also found to physically interact in HEK239T cells when overexpressed (56). However, the precise mechanisms by which Ahr and RORγt regulate IL-22 expression have yet to be elucidated. The recruitment of Ahr to the *Il22* locus requires co-expression of RORγt in both EL4 cells and primary T cells, while RORγt occupies the *Il22* locus independently of Ahr (56, 72). Furthermore, RORγt deficiency is associated with a less permissive histone modification status at the locus (72). However, forced expression of Ahr or RORγt was reported to be insufficient to induce *Il22* expression in murine naïve CD4^+^ T cells that had a non-permissive epigenetic profile at the locus (72, 103), suggesting preceding transcriptional regulation that facilitates a permissive chromatin environment independent of Ahr or RORγt. Once a permissive environment is established, RORγt may facilitate enhanced Ahr binding to *Il22* and help maintain the chromatin landscape. As noted above, Stat3 may be an initiating transcription factor that facilitates the subsequent recruitment of RORγt and Ahr to the *Il22* locus to drive its transcription.

However, Ahr and RORγt do not always act in cooperation with each other. While Ahr promotes gut homing receptor GPR15, RORγt acts to inhibit Gpr15 in murine Treg cells (**Figure 1**) (28, 29). Ahr recruitment to the *Gpr15* locus is enhanced in the absence of RORγt, and reciprocally, RORγt binding is enhanced in the absence of Ahr, suggesting competition for binding at the *Gpr15* locus between Ahr and RORγt. The inhibition of Ahr binding at the *Gpr15* locus by RORγt is dependent on RORγt’s DNA binding domain, and a physical interaction between Ahr, RORγt and Foxp3 has been detected (29). Nonetheless, the exact mechanisms of Ahr and RORγt antagonism at the *Gpr15* locus have yet to be determined. Loss of Ahr expression in Treg cells had no impact on chromatin accessibility at Ahr-bound regions at the *Gpr15* locus (29), suggesting that Ahr and RORγt’s competition at the locus may be independent of chromatin accessibility, but does not rule out the possibility that histone modifications are altered. Furthermore, murine RORγt-deficient Th17 cells also significantly upregulated Gpr15 expression, and co-transduction of Ahr and RORγt in Th0 cells reduced Gpr15 expression as compared to Ahr transduction alone (29), ruling out the possibility that Ahr and RORγt antagonism at the locus relies on the presence of Foxp3. RORγt has been implicated in mediating enhancer-promoter looping of a conserved non-coding sequence (CNS) to the *Il17* and *Il17f* promoter to drive transcription potentially through the recruitment of histone modifying enzymes including p300 and JMJD3 (104). Thus, it is possible that RORγt inhibits enhancer-promoter looping at the *Gpr15* locus, while Ahr may act to promote looping at the locus. Chromosome conformation capture assays, such as H3K27ac HiChIP, may help elucidate the precise epigenetic mechanisms of Ahr and RORγt antagonism at the *Gpr15* locus.

### Ahr and Foxp3

Whether Ahr directly regulates Foxp3 expression remains unclear. In *in vitro* studies using human cells, Ahr activation induces Foxp3 expression. However, this regulation is in part indirect, as Ahr activation promoted TGFβ signaling and facilitated Smad1 binding to the *Foxp3* locus (22). In addition, in human CD4^+^ T cells, administration of an Ahr ligand ITE drove *Foxp3* expression (23). However, in mice, Ahr may not be required for maintaining Foxp3 expression. Ahr ablation specifically in Treg cells had no impact on *Foxp3* expression, and the methylation and chromatin accessibility status at the *Foxp3* locus is unaltered (27). It remains a possibility that Ahr may differentially regulate *Foxp3* expression in mice and humans, or may reflect differences in Ahr function *in vivo* versus *in vitro*.

While Ahr may not directly regulate *Foxp3* expression, it is essential for ensuring Foxp3 function at specific loci. Recent studies in mice have highlighted the role for Ahr in promoting murine Treg cell homing to the intestines by driving Gpr15, a gut homing receptor (**Figure 1**) (28, 29). ChIP-seq analysis revealed that Ahr and Foxp3 bound to similar regions downstream of *Gpr15*, and Foxp3 shRNA inhibited *Gpr15* expression *in vitro.* However, Foxp3’s ability to drive *Gpr15* expression was dependent on Ahr, and intriguingly, Foxp3 binding was dispensable for its ability to promote *Gpr15* expression, while Ahr DNA binding activity was required. Ahr and Foxp3 physically interacted through Ahr’s Per-ARNT-SIM and basic helix-loop-helix domain, and the leucine-zipper and forkhead domains of Foxp3 (29). Thus, transcriptional regulation of the *Gpr15* locus by Foxp3 is dependent on its physical interaction with Ahr and subsequent direct DNA binding to the locus by Ahr. Intriguingly, Ahr activation by exogenous TCDD administration significantly enhanced *Gpr15* mRNA in Tr1 cells, which lack Foxp3 expression, suggesting that Ahr may regulate *Gpr15* in Tr1 cells and that Ahr does not require Foxp3 to drive *Gpr15* in other cell subsets (31). However, Ahr-deficient CD8^+^ T cells have unaltered expression of Gpr15, demonstrating cell type dependent regulation of Gpr15 by Ahr (25). Overexpression of Ahr in Th0 cells was sufficient to enhance Gpr15 protein *in vitro*, while co-transduction with Foxp3 enhanced Gpr15 (29). Notably, cooperation between Ahr and Foxp3 to promote transcription in Foxp3^+^ Treg cells may be a rare event at other loci. Deletion of Ahr solely in murine Treg cells led to relatively few transcriptional changes and were largely related to intestinal homing (27). Direct targets of Foxp3, including *Il2* and *Il2ra* (105) were unchanged in Ahr-deficient Treg cells. Given the dispensable nature of Foxp3’s DNA binding domain to promote *Gpr15* expression (29), the synergistic transcriptional regulation of *Gpr15* relies on prior Ahr genomic occupancy. Given Ahr’s ability to recruit RNA polymerase II to bound promoter regions (106), complexing with Foxp3 may enhance this recruitment effect thus explaining the synergistic effect of Foxp3 and Ahr transduction in driving *Gpr15* in Th0 cells (29). Of note, a separate study demonstrated that Ahr and GATA3 could synergistically drive *Gpr15* expression in luciferase assay (28). However, this cooperation needs to be further corroborated *in vivo.*

### Ahr and Aiolos

Ahr sustains the expression Aiolos in both Treg and Th17 cells to silence IL-2 expression (22, 34). Ahr ligand administration enhanced Aiolos binding to the *IL2* promoter in human Treg cells (22), and, as noted previously, Ahr directly binds to the *Ikzf3* (encoding Aiolos) locus to synergistically drive *Ikzf3* expression in conjunction with Stat3 in murine Th17 cells to promote Th17 cell differentiation (**Figure 1**) (34). Ahr also regulates Aiolos expression in ILC2s, reflecting a shared transcriptional target of Ahr between both ILC and T cell lineages (**Figure 1**). As noted earlier, Ahr restricted IL-33 signaling and type 2 cytokine production in gut ILC2s (52), but not lung ILC2s (54), demonstrating tissue specific effects on Ahr transcriptional activity. Furthermore, while Ahr was expressed at low levels in the lung at steady state (52), IL-33 treatment revealed a role for Ahr in lung ILC2s under certain conditions (54). Intriguingly, in the absence of IL-33 treatment, Ahr cell intrinsically regulated Aiolos expression solely in intestinal ILC2s, and Aiolos expression was low in lung ILC2s (94). Mechanistically, Ahr regulates *Ikzf3* through two intestine-specific open chromatin regions (94). In conjunction with Ahr’s inability to drive Aiolos expression in lung ILC2s, these results suggest that Ahr may rely on tissue-specific chromatin remodelers to regulate *Ikzf3.* Furthermore, the gut-specific restriction of IL-33 signaling by Ahr in ILC2s may be reliant on the presence of Aiolos. Establishing the impact of dual Ahr and Aiolos deficiency on tissue-specific regulation of ILC2 may help elucidate the relationship between Ahr and Aiolos in regulating ILC2 in different tissues.

### Ahr and Hif-1α

Ahr and Hif-1α have an antagonistic relationship in driving murine Tr1 cell differentiation (**Figure 1**) (97). This antagonism is in part explained through competition of Ahr and Hif-1α to bind to Arnt, although Arnt is in excess in most cell types. *In vitro*, Hif-1α expression is induced by extracellular ATP (eATP) and inhibits Tr1 cell differentiation by sequestration of Arnt from Ahr and induces Ahr proteasomal degradation. Reciprocal Ahr/Arnt interaction also triggers Hif-1α proteasomal degradation. To limit Hif-1α’s suppressive activity, Ahr induces expression of the ectonucleotidase *Entpd1*, which converts eATP into adenosine monophosphate (AMP) and reduces Hif-1α expression. However, Hif-1α-mediated suppression of Tr1 cell differentiation only occurs later during Tr1 cell differentiation. Immediately following activation when Ahr is not expressed, Hif-1α promotes Tr1 cell differentiation by sustaining aerobic glycolysis. At later timepoints, Ahr is subsequently required to sustain the metabolic program of Tr1 cells. The regulatory mechanisms that facilitate this transcriptional switch have yet to be fully elucidated, though Stat3 may facilitate this switch *via* two mechanisms. First, Stat3 can bind to the *Ahr* promoter in Tr1 cells and directly promote *Ahr* expression. Second, it can drive expression of *Entpd1* in Tr1 cells in luciferase assay in the absence of Ahr, thereby limiting eATP-mediated induction of Hif-1α and favoring Ahr stability (97). Experimental validation through the knockdown of Stat3 in conjunction with *Entpd1* may elucidate the specific contribution of Stat3 in driving the metabolic switch of Tr1 cells from Hif-1α to Ahr dependence. In addition, whether Hif-1α and Ahr antagonism exists, particularly in intestinal ILC3s and CD8^+^ TILs, is of particular interest. Both Ahr and Hif-1α promote ILC3 ontogeny and effector function (55, 56, 107, 108). Furthermore, Ahr and Hif-1α promote CD8^+^ TIL effector function (35, 109). Careful consideration of the transcriptional and post-translational antagonism between Ahr and Hif-1α will be essential in elucidating their relationship in other T cells and ILCs.

### Ahr and Ikaros

Ikaros can regulate Ahr expression and function in a cell type and gene-dependent manner. Ikaros restricts postnatal ILC3s in part through suppression of Ahr function (110). It can physically interact with Ahr in primary ILC3s, as revealed by proximity ligation assay, and this interaction suppresses dimerization of Ahr/Arnt and reduces Ahr binding to target genes (110). However, in T cells, Ikaros may promote expression of Ahr, as T cells lacking Ikaros, or expressing Ikaros lacking the DNA-binding zinc finger 4 domain downregulates Ahr (99, 111). Ikaros directly binds to the *Ahr* locus as revealed by ChIP assay in Th17 cells and is associated with permissive histone modifications at those binding sites, suggesting Ikaros may sustain *Ahr* expression through direct transcriptional activation and chromatin remodeling in Th17 cells (**Figure 1**) (111). Intriguingly, despite sustaining Ahr expression, cells expressing Ikaros lacking the DNA binding domain significantly upregulated IL-22 in an Ahr-dependent manner (99). Given the requirement for the DNA binding domain in facilitating Ikaros’ interaction and suppression of Ahr/Arnt dimerization in ILC3s (110), Ikaros may similarly suppress Ahr activity in Th17 cells, despite also sustaining Ahr expression in those cells as well. However, not all Ahr target genes were impacted in Th17 cells expressing the Ikaros DNA binding mutant, suggesting a gene-dependent regulation of Ahr activity by Ikaros (99). The gene-specific repression of Ahr function by Ikaros may be reliant on additional Ahr-interacting partners occupying distinct Ikaros-bound loci. Given the abundance of *in vitro* differentiated Th17 cells, biochemical and epigenetic studies to systematically identify different Ahr and Ikaros binding partners may be an attractive avenue to pursue.

### Ahr and Blimp1

Blimp1 has been proposed as a master regulator of driving immune cell residency in a diverse range of immune cells including murine CD8^+^ T cells, CD4^+^ T cells and NK cells (112, 113). In adoptively transferred Ahr-deficient CD8^+^ T cells, *Prdm1* (encoding Blimp1) mRNA is significantly reduced in T_RM_ cells following *Listeria* infection (35). Further, Ahr ChIP-seq in *in vitro* differentiated T_RM_-like cells revealed direct binding by Ahr to the *Prdm1* locus, suggesting that *Prdm1* may be downstream of Ahr in promoting genes that facilitate tissue residency in CD8^+^ T cells. Furthermore, *in vitro* rescuing experiments by transduction of Blimp1 into Ahr-deficient CD8^+^ T cells rescued impaired T_RM_-like cell differentiation in the absence of Ahr. While *in vitro* over-expression assays likely due not fully recapitulate the *in vivo* mechanisms of regulation of the residency program by Ahr and Blimp1, the role for Ahr in driving residency programs in CD8^+^ T cells is of particular interest given Ahr’s high expression particularly in non-lymphoid tissues, where many cells remain resident. Indeed, Ahr promotes intestinal homing of Treg cells (27, 31) and is essential for maintenance of resident ILC3s in the intestine (55, 56). In addition, while Blimp1 is essential for T_RM_ cell differentiation, it is also highly expressed in effector CD8^+^ T cells found in the blood (114) and thus is not solely involved in driving the residency program. Whether Ahr regulates the residency program, *via* Blimp1 or otherwise, in other immune cells remains an open question. Careful dissection of common transcriptional programs regulated by Ahr across broad tissues and cell types may reveal a common theme across immune cells in Ahr maintaining immune cell residency programs.

### Self-regulation of Ahr expression

Recent evidence suggests that Ahr regulates its own expression in a cell-type specific manner and involves selective chromatin remodeling at the *Ahr* locus (**Figure 1**) (52). In gut-ILC2, a specific region of chromatin downstream of *Ahr* (“peak 6”) is accessible, while it remained inaccessible in other Ahr-expressing populations including gut-ILC3s and gut-Treg cells. Intriguingly, in the absence of Ahr, peak 6 exhibited reduced chromatin accessibility in gut-ILC2s and Ahr acted to recruit the transcription factors Gfi1 and Gata3 to the peak 6 region. These data demonstrate that Ahr promotes its own transcription in a cell-type specific manner *via* a positive-feedback loop (52). Follow up studies specifically ablating peak 6 *in vivo* may help clarify its role in Ahr’s self-regulation.

## Conclusion

Ahr has emerged as an essential regulator of a multitude of immune cells. However, its broad expression pattern and the diverse functions of Ahr across both different immune cells and organs make therapeutics targeting of Ahr challenging. Furthermore, the transcriptional regulatory networks vary widely across different cell types and organs. As such, it is essential to understand not only the cell-intrinsic functions of Ahr, but to understand its context-dependent relationship with other transcription factors. Recent advances in low to single-cell mass spectrometry will elucidate Ahr-partners in rare immune cells (80, 81). Utilization of technologies such as single-cell ATAC-seq and RNA-seq will help elucidate the role of Ahr in heterogenous populations. Leveraging of low cell input assays, such as CUT&RUN (115), will be essential to identify Ahr direct targets and how Ahr regulates the chromatin landscape in rare immune cells. In this line, whether Ahr controls 3-dimensional chromatin architecture is entirely unknown. Recent advances in assays to interrogate 3D genome structure with reduced cell input requirements will be essential to address these questions (116, 117). Utilization of these technologies in conjunction with sophisticated genetic models to modulate Ahr in tandem with other transcription factors will be required to delineate cell-type specific relationships at the chromatin level. Such mechanistic understandings may elucidate novel therapeutics to target Ahr, or Ahr partners, in a cell-type specific manner for disease prevention and treatments.

## Author contributions

EH wrote the manuscript. LZ supervised the research and edited the manuscript. All authors contributed to the article and approved the submitted version.
